# Epidemiological yield and cost-effectiveness of non-targeted community HIV screening in a low-prevalence district: a prospective quasi-experimental study

**DOI:** 10.3389/fpubh.2026.1882126

**Published:** 2026-07-14

**Authors:** Yu Luo, Lu Liu, Hong Li, Xiyue Fan, Xinglan Tan, Wenting Yang

**Affiliations:** Beibei Center for Disease Control and Prevention, Chongqing, China

**Keywords:** cost-effectiveness, expanded screening, HIV/AIDS, known positive, mobile populations

## Abstract

**Objective:**

To evaluate the epidemiological yield, cost-effectiveness, and mechanisms of diagnostic cascade attrition of active, expanded community-based HIV screening in a low-prevalence region, and to contextualize the regional management burden.

**Methods:**

A prospective, non-randomized, quasi-experimental study was conducted (August–December 2025) across six demographically matched sub-districts. Three implemented active community screening (intervention); three maintained routine facility-based testing (control). A deduplicated dual-track database mapped the diagnostic cascade, supplemented by a 7-month longitudinal cohort to assess regional disease burden. Micro-costing determined fixed and variable costs from a health-system perspective. The Number Needed to Screen (NNS), Cost Per New Diagnosis (CPND), and Incremental Cost-Effectiveness Ratio (ICER) were calculated, supported by Propensity Score Matching (PSM), permutation tests, and deterministic sensitivity analyses.

**Results:**

Among 17,305 unique participants (intervention: 14,434; control: 2,871), the intervention expanded coverage in the 35–49 age cohort (35.0% vs. 25.5%, *p* < 0.001). However, only one true new HIV diagnosis occurred in the intervention arm (yield: 0.0069%), showing no statistical difference from the control (0%, Fisher’s exact test *p* = 1.000), a finding robustly confirmed by PSM and permutation tests. The overall NNS was 14,434, remaining undefined (>1,250 and >5,055) for the highly prioritized 18–49 age cohorts. Longitudinal cohort data revealed a stark demographic mismatch: 76.0% of true local incident cases were aged ≧50 years, and the regional management burden was increasingly driven by migrant populations rather than local transmission. Notably, 80.0% (8/10) of reactive initial screens in the intervention group were previously diagnosed individuals, predominantly older males (87.5% male; 75.0% aged 50–64). While temporal correlation was not statistically significant (Spearman’s *ρ* = 0.564, *p* = 0.322), the absolute metrics highlight substantial known-positive interference. Consequently, the intervention yielded an ICER of 504,886 CNY per additional diagnosis, remaining above the willingness-to-pay threshold even under extreme sensitivity scenarios.

**Conclusion:**

In epidemiologically saturated settings, non-targeted community screening yields profound diminishing marginal returns. Material incentives inadvertently capture previously diagnosed older males who undergo repeated testing, resulting in substantial resource redundancy rather than identifying occult infections. To optimize cost-effectiveness, public health strategies must implement digital pre-screening interception mechanisms and strategically reallocate funds toward key-population friendly services and peer-driven interventions.

## 1 Introduction

Human Immunodeficiency Virus (HIV) infection, primarily a sexually transmitted chronic infectious disease, remains a major global public health challenge (1). Although the global incidence of new HIV infections is generally declining, the epidemic continues to be characterized by uneven geographical distribution and concentration within key populations. In China, following years of comprehensive prevention and control efforts since the first reported case, the overall epidemic has been maintained at a low prevalence level, with the national population infection rate consistently below 0.1%. However, clustered epidemics persist among specific high-risk populations and in localized areas (2). Against this backdrop, national and regional authorities have successively issued policies to curb HIV transmission. Notably, guidelines on expanding testing mandate the implementation of active community-based screening in key regions. Combining facility-based testing with community mobilization, these policies recommend targeted testing for individuals aged 18–64 years in areas with an overall population prevalence of ≥0.5%, and for those aged 35–64 years in areas with a prevalence of 0.25–0.49%.

From the perspective of infectious disease control theory, the core logic of HIV prevention and treatment lies in “early detection, early diagnosis, and early treatment.” Extensive research has confirmed that initiating antiretroviral therapy (ART) as early as possible not only significantly reduces individual morbidity and mortality but also achieves “Treatment as Prevention” (TasP) by suppressing viral loads, thereby mitigating secondary transmission (3, 4). Therefore, increasing testing coverage and reducing diagnostic delays are critical prerequisites (5, 6). HIV testing strategies have evolved from passive, facility-based testing to more diversified and accessible modalities. Early voluntary counseling and testing (VCT) targeting high-risk groups has gradually expanded to encompass inpatient and outpatient screening, blood product screening, and prenatal testing (7). More recently, community-based outreach testing (8, 9), HIV self-testing (HIVST), and targeted testing integrated with other screening programs (10–12)have been deployed, all aiming to enhance testing coverage and facilitate early detection.

Although China remains an overall low-prevalence country and most districts and counties have achieved high testing and treatment coverage, there is a dearth of empirical evidence supporting the epidemiological yield and health economic value of conducting further large-scale, active community screening among the general population. Particularly under the rigid resource constraints faced by local Centers for Disease Control and Prevention (CDCs) and community healthcare facilities, expanding the screening scale when the new positivity rate is extremely low may lead to the repeated testing of previously diagnosed individuals and the unnecessary mobilization of low-risk populations. These phenomena can result in substantial resource waste and reduced allocative efficiency. Existing domestic and international cost-effectiveness studies on HIV testing are predominantly based on mathematical modeling or focus primarily on high-risk populations (13–17). Real-world, prospective controlled studies focusing on general community populations in low-prevalence areas remain scarce. In particular, there are no systematic reports quantitatively evaluating the impact of “interfering reactive results” (i.e., known positives) on economic indicators during expanded screening. To bridge this gap, this study utilizes a real-world, match-control design in a low-prevalence district in Southwest China. By comparing the strategy of active, expanded community-based HIV screening with the routine passive facility-based testing model, this study systematically evaluates their comparative epidemiological yields and health economic cost-effectiveness.

## 2 Materials and methods

### 2.1 Study design and setting

A prospective, non-randomized, quasi-experimental study was conducted from 01/08/2025 to 31/12/2025. A randomized controlled trial (RCT) was not feasible due to the high risk of intervention contamination across administrative boundaries during community-level mobilization. The study was conducted in a district of Southwest China, a region historically characterized by a high HIV burden that is currently transitioning to a low-prevalence state, nearing the UNAIDS 95–95-95 targets. The five-month study duration aligned with the local Center for Disease Control and Prevention (CDC) fiscal-year budget cycle for community outreach, ensuring a realistic evaluation of rigid resource constraints. Six pilot sub-districts were purposively selected to align with the universal HIV testing policies issued by higher administrative authorities. The intervention sub-districts were designated based on local epidemic control imperatives and key prevention priorities. To minimize selection bias, the control sub-districts were carefully matched prior to selection based on four comprehensive criteria: the overall population HIV/AIDS prevalence, local economic development levels, the age structure of permanent residents, and urban–rural geographical characteristics. Detailed baseline demographics, target population sizes, and historical HIV prevalence demonstrating the equivalence of the matched sub-districts are provided in Table 1.

**Table 1:** Baseline demographic and epidemiological characteristics of the selected sub-districts prior to the intervention.

Matched pairs	Sub-district	Assignment	Target age group	Total population	Target population	Age structure proportion (<18: 18–34: 35–49: 50–64: ≥65)	Historical HIV prevalence*
Pair1	T01	Intervention	18–64	18,676	11,563	8%: 12%: 18%: 31%: 30%	0.571%
	T04	Control	18–64	40,285	25,007	9%: 12%: 18%: 32%: 29%	0.552%
Pair2	T02	Intervention	35–64	33,287	16,522	10%: 13%: 22%: 28%: 27%	0.418%
	T05	Control	35–64	57,446	25,434	17%: 19%: 20%: 24%: 20%	0.332%
Pair3	T03	Intervention	35–64	22,950	10,754	9%: 14%: 21%: 26%: 30%	0.464%
	T06	Control	35–64	7,335	3,258	8%: 11%: 12%: 33%: 36%	0.355%

*Historical HIV prevalence refers to the overall population infection rate recorded prior to the initiation of the study in July 2025.

### 2.2 Intervention and control strategies

The six sub-districts were assigned to two study arms:

Active Expanded Screening (Intervention Arm): Implemented in three sub-districts (one high-prevalence area targeting ages 18–64; two mid-prevalence areas targeting ages 35–64). Executed by community health centers and grassroots coordinators, this strategy mobilized populations via pop-up sites, educational materials, and small material incentives (e.g., daily necessities valued at 10–15 CNY). Coordinators received standardized training on testing protocols and data recording prior to implementation.

Routine Passive Testing (Control Arm): Implemented in the three matched sub-districts. This arm relied exclusively on routine, facility-based provider-initiated testing and counseling (PITC) [e.g., preoperative screenings, inpatient testing, and voluntary counseling and testing (VCT) clinics], with no additional community mobilization.

### 2.3 Data integration, exclusions, and cohort definition

To distinguish between systematic testing volume and actual epidemiological coverage, a dual-track dataset was constructed. Records with missing or invalid identification numbers [accounting for <2% of total encounters, assumed missing completely at random (MCAR)] and those outside the target age ranges were excluded without imputation.

Encounter-level dataset: Retained all valid testing encounters, including repeated tests by the same individual, to calculate overall input costs and physical testing redundancy.

Individual-level dataset: Deduplicated using exact matching of valid identification numbers. For individuals with multiple encounters, the earliest reactive (positive) screening record was prioritized; otherwise, the chronologically earliest negative record was retained.

Longitudinal Cohort Dataset: To contextualize the regional disease burden and justify the target age groups, an active management cohort comprising monthly survival data of all registered HIV cases in the district from January to July 2025 was analyzed. The longitudinal cohort data for this study were formally accessed on 31/07/2025. Within this cohort, local incident cases were defined as patients newly added during the follow-up period whose interval between first diagnosis and entry into the local system was ≤ 1 month, representing cases screened locally and managed on-site. Incoming migrants were defined as patients newly added during the follow-up period whose first diagnosis occurred > 1 month before entry into the local system. The 1-month threshold was strategically selected to account for standard administrative protocols, as the mandatory reporting, confirmatory testing, and local file-establishment processes are typically completed within a 30-day window. To rigorously address concerns regarding potential misclassification bias, a sensitivity analysis was conducted by extending this threshold to 3 months. This group represents individuals carrying prior confirmed medical records who newly migrated into the local management system due to inter-regional mobility and change of residence.

### 2.4 Outcome measures and the diagnostic cascade

Adhering to national HIV testing standards, clinical evaluation nodes along the diagnostic cascade included:

Known positives: Individuals with a reactive initial screen who were confirmed via the National Comprehensive HIV/AIDS Prevention and Control Information System to have a pre-existing diagnosis or an active treatment record prior to the current screening.

New diagnoses: Individuals with a reactive initial screen, subsequently confirmed positive by supplementary testing, with no historical record in the national system.

Testing redundancy rate: Calculated as (Total testing encounters - Unique individuals screened)/Total testing encounters × 100%.

Known-positive interference rate: The proportion of known positives among all individuals with a reactive initial screen.

Number needed to screen (NNS): The total number of unique individuals required to be screened to identify one true newly diagnosed HIV case (18).

### 2.5 Health economic evaluation

A bottom-up micro-costing approach was applied from a health system perspective, explicitly excluding indirect societal costs (e.g., patient time, transportation) to reflect the actual budget allocations and rigid fiscal constraints of local CDCs. Fixed costs included testing reagents and the prorated cost of routine medical personnel time. Variable costs (incurred exclusively in the intervention arm) encompassed mobilization funds, incentives, and supplementary operational expenses. Primary economic indicators were the Cost Per New Diagnosis (CPND) and the Incremental Cost-Effectiveness Ratio (ICER), calculated as:


$$\text{ICER}=(\cos t_{\mathrm{int}}-\cos t_{\text{ctrl}})/({\text{Yield}}_{\mathrm{int}}-{\text{Yield}}_{\text{ctrl}})$$

Regarding the willingness-to-pay (WTP) threshold, recent analyses suggest that it should be adjusted based on specific disease categories and local economic conditions, with some estimates recommending a threshold of 1.76 to 2.06 times the GDP per capita (19). However, given that many established health economic studies continue to utilize the traditional heuristic, this study selected three times the local per capita GDP (approximately 329,100 CNY, based on the local per capita GDP of ~109,700 CNY) as the WTP threshold (20, 21).

### 2.6 Statistical analysis

Data management and statistical analyses were conducted using Python (version 3.10). Categorical variables were compared using Pearson’s Chi-square test; Fisher’s exact test was applied when event counts were zero. Spearman’s rank correlation was utilized to assess the relationship between monthly testing volume and known-positive interference. Adjustments for clustering at the sub-district level were not performed due to the small number of clusters (*n* = 6), which typically leads to biased variance estimates. Consequently, a Monte Carlo permutation test (5,000 iterations) was employed to robustly estimate *p*-values while accounting for potential clustering effects. To address baseline demographic imbalances, a Propensity Score Matching (PSM) analysis was conducted, matching participants 1:1 based on age and gender using nearest-neighbor matching, followed by Fisher’s exact test on the balanced sub-cohort. Cramer’s V was calculated to evaluate the effect sizes of baseline differences. A two-sided *p* < 0.05 was considered statistically significant.

## 3 Results

### 3.1 Screening coverage and baseline characteristics

A total of 19,149 testing encounters met the inclusion criteria. Following deduplication based on valid identification numbers, 17,305 unique individuals were enrolled (intervention group: *n* = 14,434; control group: *n* = 2,871).

As detailed in Table 2, the proportion of female participants in the intervention group (56.8%) was higher than in the control group (54.7%) (*p* = 0.042); however, the effect size for this difference was negligible (Cramer’s *V* = 0.013). Regarding age distribution, the control group was predominantly concentrated in the 50–64 age bracket (68.7%). The active intervention strategy expanded coverage among younger and middle-aged adults (35–49 years), increasing their proportion to 35.0% compared to 25.5% in the control group (*p* < 0.001; Cramer’s *V* = 0.093). This demonstrates the capacity of community-based screening to broaden the demographic reach beyond routine facility-based attendees.

**Table 2:** Baseline demographic characteristics of independent individuals screened by strategy.

Demographics	Intervention arm	Control arm	$x^{2}$	*P*-value	Cramer’s V
	*n* = 14,344	*n* = 2,871			
Gender, n(%)			4.12	0.042	0.013
Male	6,236(43.2%)	1,300 (45.3%)			
Female	8,198 (56.8%)	1,571 (54.7%)			
Age group, n(%)			151.27	<0.001	0.093
18–34	1,250 (8.7%)	168 (5.9%)			
35–49	5,055 (35.0%)	731 (25.5%)			
50–64	8,129 (56.3%)	1,972 (68.7%)			

### 3.2 Diagnostic cascade and resource attrition patterns

**Figure 1:**
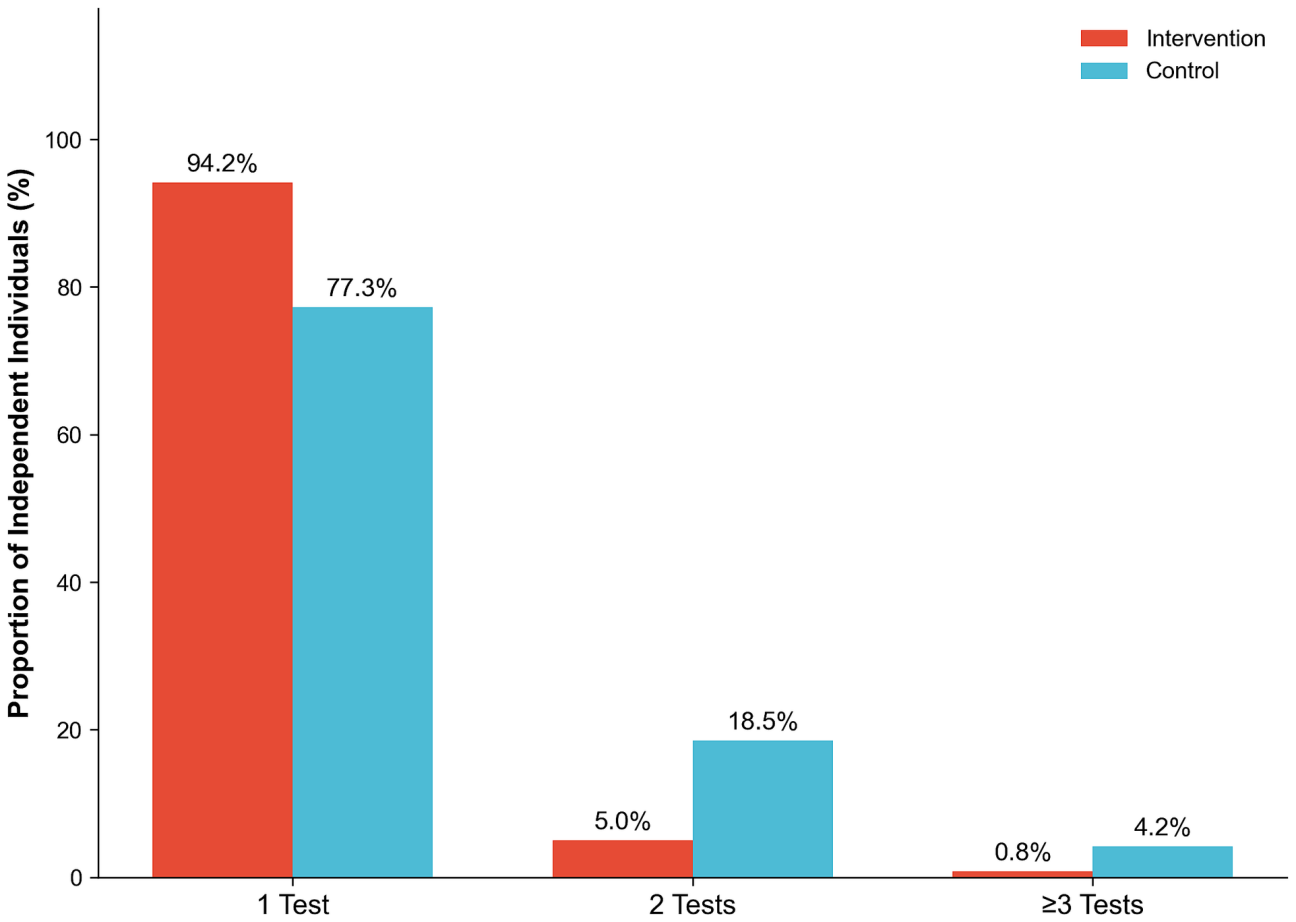
Distribution of repeat testing frequency.

Mapping the HIV diagnostic cascade revealed distinct mechanisms of resource attrition between the two arms. The control group exhibited a physical testing redundancy rate of 23.13%. Testing frequency distribution (Figure 1) indicated that 22.7% of participants in the control group underwent ≥2 tests within the 5-month period, with 4.2% undergoing ≥3 tests. This was primarily driven by compliant clinical pathways, such as repeated blood draws for inpatient and preoperative screenings.

**Figure 2:**
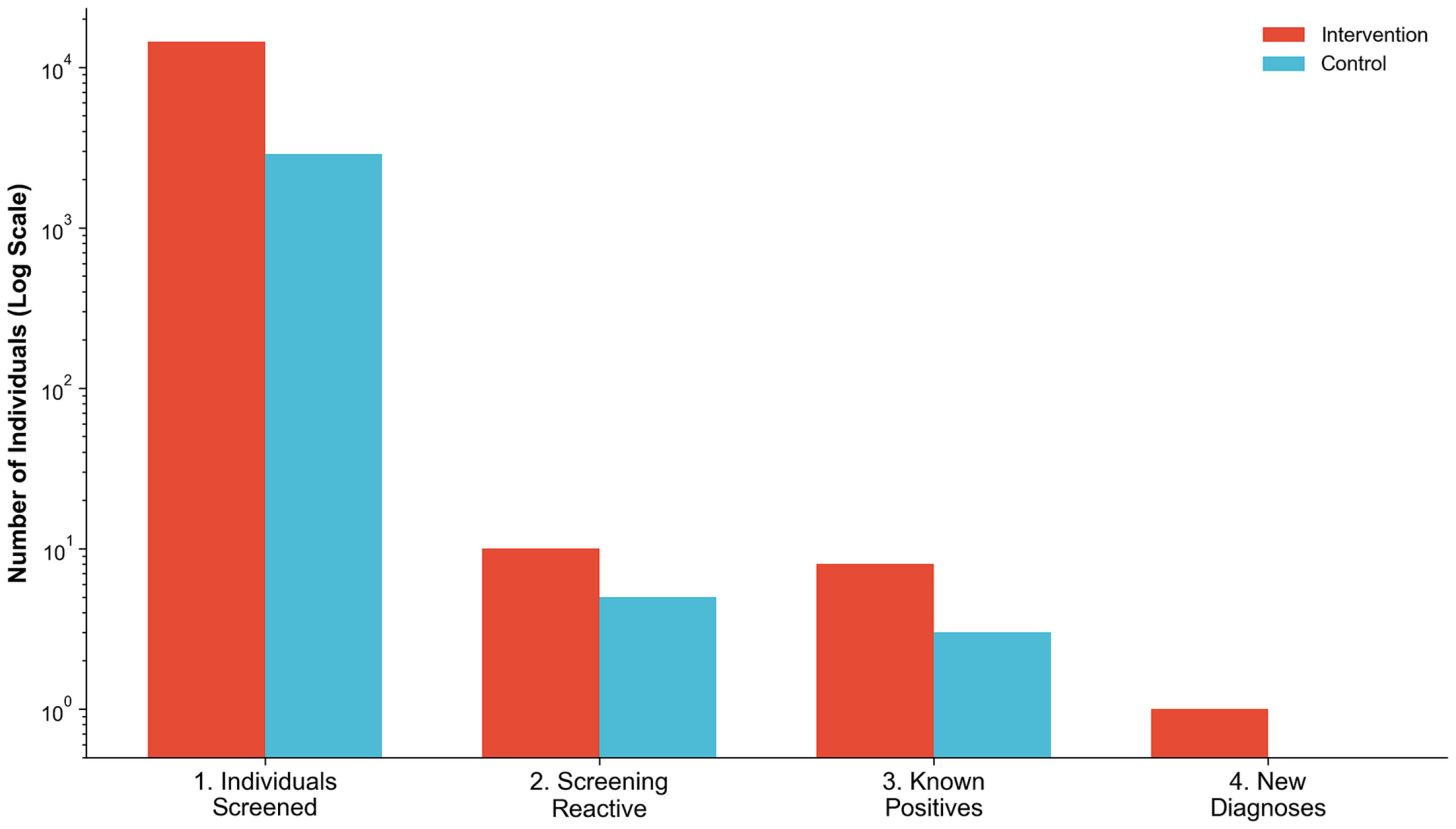
Comparison of HIV diagnostic cascade by strategy.

Conversely, while the intervention group demonstrated a lower physical redundancy rate (6.36%), it experienced substantial attrition due to known-positive interference. As illustrated in Figure 2, among the 14,434 unique individuals screened, 10 yielded reactive initial results; however, system verification confirmed that 8 were previously diagnosed HIV patients. Consequently, the known-positive interference rate in the intervention arm reached 80.00%, compared to 60.00% (3 of 5) in the control arm.

### 3.3 Subgroup analysis of interfering known positives

A demographic subgroup analysis of the 8 known-positive individuals who utilized screening resources in the intervention group indicated extreme demographic clustering (Table 3). Specifically, 7 of 8 (87.5%) were male, and 6 of 8 (75.0%) fell within the 50–64 age bracket. This suggests that this specific demographic may be particularly responsive to the material incentives provided in the community mobilization model, leading to repeated but epidemiologically uninformative testing.

**Table 3:** Demographic profile of known-positive interference in the Intervention group.

Characteristics	Total known positives (*N* = 8)	Raw numbers (n)	Proportion (%)
Gender			
Male	7		87.5%
Female	1		12.5%
Age group			
18–34	1		12.5%
35–49	1		12.5%
50–64	6		75.0%

### 3.4 Epidemiological stratification efficiency and NNS

After excluding cases lost to confirmatory follow-up (*n* = 1) and known positives (*n* = 8), only 1 true newly diagnosed positive case was identified in the intervention group. The true diagnostic yield was 0.0069%, showing no statistical difference from the control group’s yield of 0.0000% (0 of 2,871; Fisher’s exact test, *p* = 1.000). To robustly account for potential clustering, a permutation test (5,000 iterations) confirmed this non-significant difference (*p* = 1.000). Furthermore, after applying Propensity Score Matching (PSM) to adjust for baseline age and gender imbalances, a 1:1 balanced sub-cohort (*n* = 5,742) was generated. Within this matched cohort, the true diagnostic yield remained zero in both arms (Fisher’s exact test, *p* = 1.000), confirming that the lack of epidemiological yield was independent of baseline demographic confounders. The overall Number Needed to Screen (NNS) for the intervention arm reached 14,434 (Table 4).

**Table 4:** HIV diagnostic cascade and epidemiological yield by screening strategy.

Yield metrics	Intervention arm	Control arm	Notes
1. Screening workload and redundancy			
Total encounters (tests), n	15,414	3,735	–
Unique individuals, n	14,434	2,871	–
Testing redundancy rate	6.36%	23.13%	Driven by passive clinical retesting
2. Diagnostic cascade nodes			
Screening reactive (initial positive), n	10	5	–
Lost to follow-up/refused, n(%)	1(10.00%)	1(20.00%)	Proportion of initial reactives
Known positives, n	8	3	Confirmed via registry
Known-positive interference rate	80.00%	60.00%	Driven by active mobilization
True new diagnoses, n	1	0	Confirmed with no historical record
3. Epidemiological efficiency			
True yield rate	0.0069%	0.0000%	Fisher’s exact/permutation, *P* = 1.000
Number needed to screen (NNS)	14,434	>2871	Individuals screened per 1 new diagnosis

Furthermore, age-stratified efficiency analysis (Table 5) revealed that in the prioritized 18–34 (*n* = 1,250) and 35–49 (*n* = 5,055) age cohorts, the number of true new infections was zero, resulting in an undefined NNS (>1,250 and >5,055, respectively). The sole new diagnosis originated from the 50–64 age group, yielding a stratified NNS of 8,129.

**Table 5:** Age-stratified epidemiological yield and NNS in the Intervention group.

Age group	Individuals screened	Known positives	New diagnoses	Stratified NNS
18–34	1,250	1	0	>1,250
35–49	5,055	1	0	>5,055
50–64	8,129	6	1	8,129

### 3.5 Time-series trends of known-positive identification

**Figure 3:**
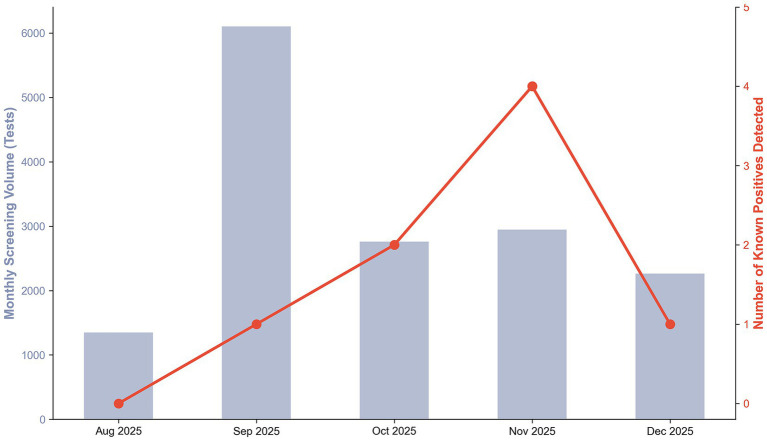
Temporal trends of screening volume and interference.

Spearman’s rank correlation analysis evaluated the dynamic relationship between monthly screening volume and the detection of known positives in the intervention arm (Figure 3). The data indicated a moderate positive correlation (*ρ* = 0.564, *p* = 0.322). As this result did not reach statistical significance, a definitive temporal association between screening volume and known-positive interference could not be established within the 5-month observation period.

### 3.6 Cost deconstruction and ICER

**Figure 4:**
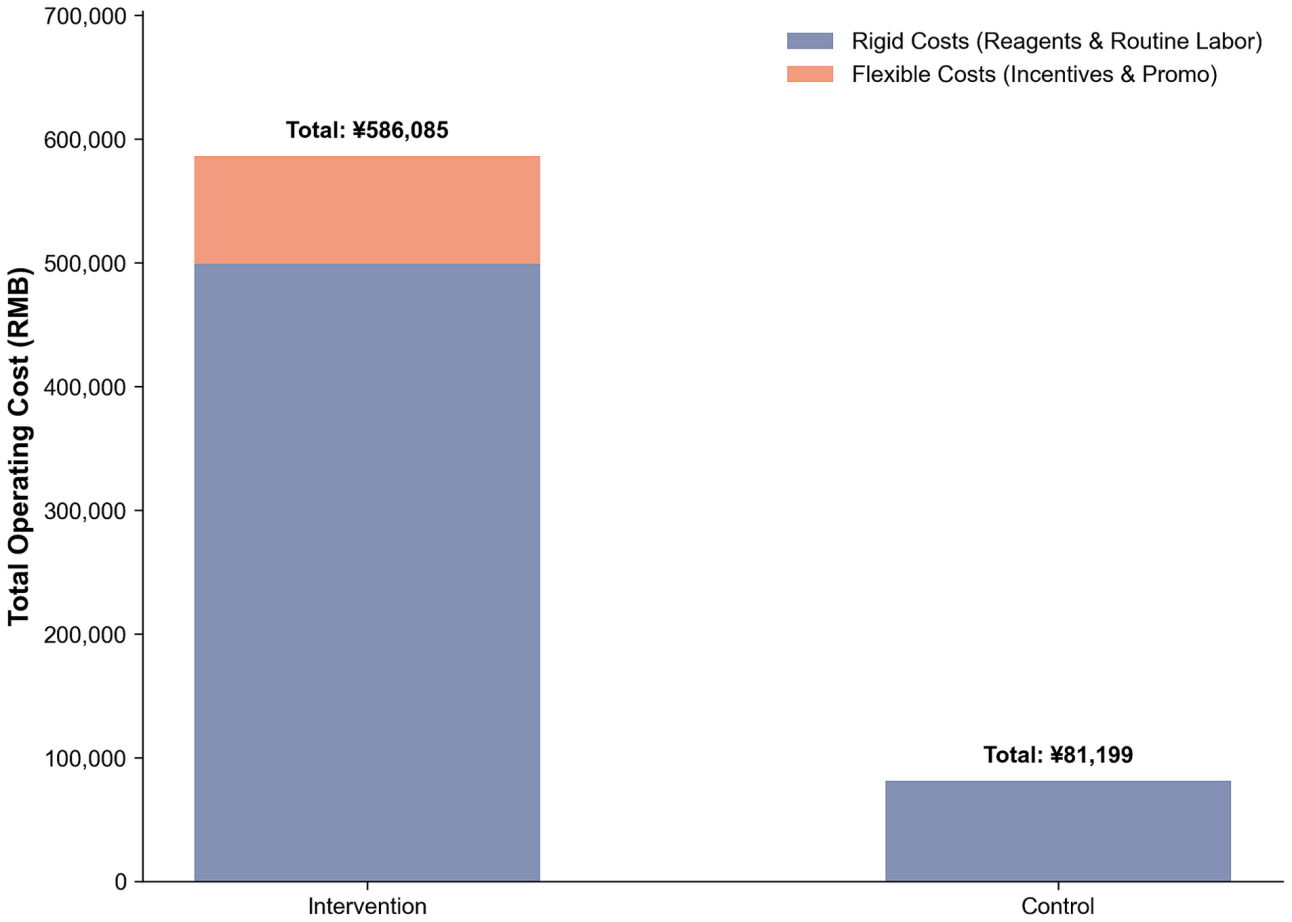
Health economic cost breakdown.

Micro-costing demonstrated a total expenditure of 81,199 CNY in the control group, 100% of which constituted fixed rigid costs (reagents and routine labor). In contrast, total expenditure in the intervention group was 586,085 CNY, comprising 498,915 CNY in fixed costs and an additional 87,170 CNY in flexible variable costs (community mobilization and incentives) (Figure 4).

The Cost Per New Diagnosis (CPND) for the intervention group was exactly 586,085 CNY. Incremental analysis revealed that for every additional new HIV diagnosis gained through active expanded screening, the health system bore an Incremental Cost-Effectiveness Ratio (ICER) of 504,886.06 CNY.

### 3.7 Sensitivity analysis

Loss to confirmatory follow-up (*n* = 1 in each arm) primarily occurred because, despite repeated prompting by healthcare staff, these individuals refused confirmatory blood draws, potentially due to HIV-related stigma or the absence of clinical symptoms. While it is highly unlikely that these individuals were previously diagnosed cases—as their historical records would have been automatically flagged by the national registry system during the initial screening—deterministic sensitivity analyses were nevertheless conducted under both optimistic and pessimistic scenarios to test the robustness of the economic outcomes. Under an optimistic scenario (assuming 100% of those lost to follow-up were true occult infections), the intervention group’s new diagnoses increase to 2, and the control group’s to 1. In this case, the incremental yield ($\Delta$Yield) remains 1, leaving the ICER solidly at 504,886 CNY. Conversely, under a pessimistic scenario (assuming all lost individuals were true negatives or, hypothetically, unrecorded known positives), the true new diagnoses remain 1 in the intervention arm and 0 in the control arm. Consequently, the incremental yield is unchanged, leaving the ICER strictly solidified at approximately 504,886 CNY.

**Figure 5:**
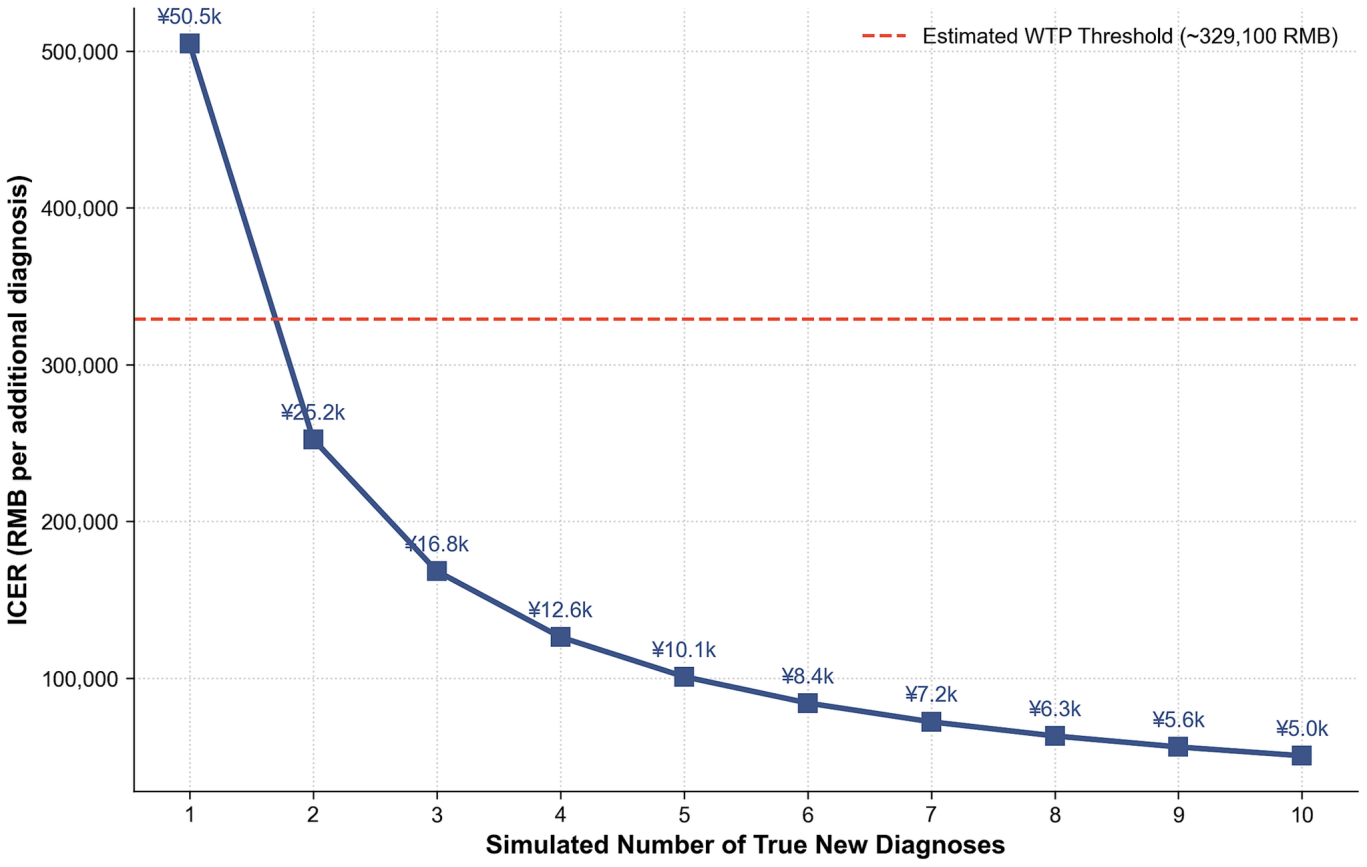
ICER sensitivity analysis.

Relaxing the assumptions to simulate the hypothetical identification of 1 to 10 occult infections in the intervention arm (assuming 0 in the control arm), the ICER trend (Figure 5) indicates that discovering 2 additional cases lowers the ICER to 252,443 CNY. Only under this simulated scenario does the ICER fall below the estimated local willingness-to-pay threshold of approximately 329,100 CNY (3 * GDP per capita). This stress test confirms that unless the epidemiological yield substantially deviates from observed reality, the non-targeted screening strategy is not cost-effective.

### 3.8 Regional disease burden and population dynamics

**Figure 6:**
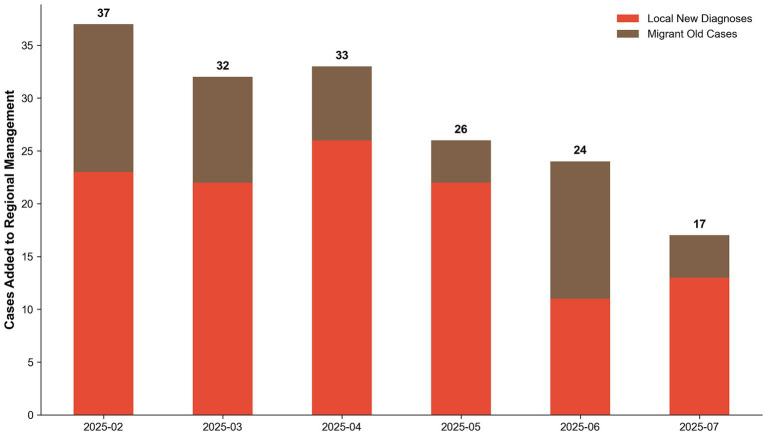
Composition of new management burden.

To contextualize the epidemiological saturation and evaluate the appropriateness of the intervention’s target age range (18–64 years), longitudinal cohort data from January to July 2025 were analyzed (Figure 6).

The data revealed a striking mismatch: among true local newly diagnosed cases across the region, 76.0% were aged ≧50 years (33.3% aged 50–64; 42.7% aged ≧65), whereas younger demographics (18–49 years) accounted for only 23.1%. Additionally, the cohort data highlighted the impact of population mobility: a substantial proportion of individuals added to the regional management registry each month were not locally incident cases, but previously diagnosed migrant cases relocating to the district. This indicates that the true regional management burden is increasingly driven by population influx rather than local transmission networks.

## 4 Discussion

Research confirms that in low-prevalence regions with a well-defined epidemiological baseline, non-targeted expanded screening frequently encounters profound diminishing marginal returns. In high-prevalence areas or among specific key populations, community-based expanded screening can significantly improve testing accessibility and diagnostic yield, demonstrating favorable cost-effectiveness under defined threshold conditions (22, 23). However, when a region’s overall prevalence is low and treatment coverage is already high, the pool of undiagnosed individuals naturally depletes, causing the financial investment required to detect each new case to increase substantially. Failing to adapt testing strategies to this epidemiological shift inevitably compromises public health efficiency (24).

### 4.1 Epidemiological saturation and demographic mismatch

When the undiagnosed rate within a target population remains low, the cost-effectiveness of large-scale HIV screening deteriorates sharply. The field data from this study strongly corroborate this projection. Despite mobilizing over 14,000 individuals in the intervention arm, the true new diagnostic yield was 0.0069%. The exceptionally high Number Needed to Screen (NNS) of 14,434 far exceeds universally reported figures for general population screening internationally (22, 23, 25–27). This profound inefficiency contrasts with the high yield typical of targeted screening among specific populations (26, 27).

Given the absence of a statistical difference in new positive rates between the two arms (Fisher’s exact test, *p* = 1.000), it can be inferred that the HIV epidemic among the general population in this region has reached a saturation point. Furthermore, the longitudinal cohort analysis revealed a critical demographic mismatch: while the community mobilization primarily targeted and successfully recruited younger demographics (ages 18–49), 76.0% of true local incident cases were aged ≧50 years. Additionally, a substantial share of the regional management burden stemmed from previously diagnosed migrant populations relocating to the district, rather than local transmission. Consequently, volume-driven strategies that rely solely on “expanding the denominator” within the general local population are rendered largely ineffective (24).

### 4.2 Known-positive interference and the potential “siphon effect”

A notable observation of this study is the disproportionate resource attrition caused by repeated testing among previously diagnosed individuals. While a definitive temporal association between screening volume and interference could not be statistically established (*ρ* = 0.564, *p* = 0.322), the absolute attrition metrics reveal a stark reality: 80.0% of initial reactive screens in the intervention arm were contributed by known positives. Furthermore, the extreme demographic clustering of these individuals—predominantly older males (87.5% male; 75.0% aged 50–64)—strongly suggests a potential “siphon effect,” wherein this specific subgroup may be particularly responsive to the material incentives provided during community mobilization.

The >87,000 CNY expended on community mobilization and promotional incentives in the intervention arm was intended to attract occult populations (28, 29). Instead, it inadvertently captured a specific subset of known positives who underwent repeated testing. The emergence of this phenomenon highlights a systemic flaw in grassroots implementation: the lack of effective on-site identity verification. This effect not only consumes fixed reagent costs but also exacerbates the workload of local HIV prevention personnel with futile follow-up tracing.

### 4.3 Policy shift: digital interception and resource reallocation

The ICER for implementing active community screening exceeded 504,000 CNY, severely breaching the estimated willingness-to-pay (WTP) threshold of approximately 329,100 CNY (calculated as three times the local GDP per capita). Confronted with these prohibitively high diagnostic costs, it is imperative to tailor testing strategies to local epidemiological realities. Healthcare administrations should reconsider using crude “testing coverage” as a rigid, singular key performance indicator (KPI).

To prevent incentive-driven redundancy, future community initiatives must integrate digital pre-screening exclusion mechanisms—utilizing secure identity verification against national registries to intercept known positives on-site. Furthermore, the substantial funding currently allocated to non-targeted community sweeps should be reallocated toward high-yield, cost-effective precision targets (15, 30). Maximizing the utility of HIV prevention resources requires a strategic pivot toward modalities such as HIV self-testing (HIVST), peer-driven interventions, key-population friendly services, and partner notification services (PNS), ensuring these approaches are implemented carefully to avoid exacerbating stigma (13, 31–36).

### 4.4 Limitations

This study is subject to several limitations. First, the sample was derived from a specific municipal district; its epidemiological baseline and population dynamics may not be fully representative of the highly heterogeneous low-prevalence regions nationwide. Second, the 5-month study duration was relatively short, though this deliberately mirrored the rigid fiscal-year budget cycles under which local CDCs operate. Consequently, potential seasonal variations in testing behaviors—such as decreased mobility during winter or year-end project settlements—could not be fully assessed, limiting generalizability. Third, indirect costs (e.g., patient time, transportation) were excluded; however, this health-system perspective was explicitly chosen to reflect the actual budget constraints of public health departments. Fourth, due to the small number of sub-districts (n = 6), clustering effects could not be reliably adjusted for using mixed-effects models, however, a permutation test was utilized to mitigate this analytical constraint. Finally, the diagnostic status of two individuals lost to follow-up could not be tracked longitudinally. Nevertheless, the conservative, extreme-value sensitivity analysis model employed in this study confirms that minor data perturbations cannot overturn the core macroscopic conclusion: non-targeted blanket screening is not cost-effective in this setting.

## 5 Conclusion

In regions characterized by low undiagnosed HIV rates and established prevention coverage, implementing non-targeted active community screening yields profound diminishing marginal returns (NNS > 14,000). Furthermore, such volume-driven strategies inadvertently induce incentive-driven repeated testing, disproportionately utilizing resources on previously diagnosed older males rather than identifying occult infections. An Incremental Cost-Effectiveness Ratio (ICER) exceeding 504,000 CNY per additional diagnosis confirms that blanket mass mobilization is not cost-effective in this epidemiological context. To optimize public health resource allocation, health administrations should transition away from crude screening-volume metrics. Funding must be strategically redirected toward implementing digital pre-screening interception mechanisms and fostering targeted, key-population friendly services and peer-driven interventions.

## Data Availability

The raw data supporting the conclusions of this article will be made available by the authors, without undue reservation.
